# Mean Tip Apex Distance in Patients undergoing Dynamic Hip Screw Fixation for Pertrochanteric Fractures without using Traction Table: A Descriptive Cross-sectional Study

**DOI:** 10.31729/jnma.7766

**Published:** 2022-12-31

**Authors:** Subhash Regmi, Rajesh Kumar Chaudhary, Ishor Pradhan, Bibek Banskota, Amit Joshi

**Affiliations:** 1 Department of Orthopedics, B&B Hospital, Gwarko, Lalitpur, Nepal

**Keywords:** *fracture fixation*, *hip fractures*, *operating tables*

## Abstract

**Introduction:**

Dynamic Hip Screw fixation has shown to be equally effective compared to cephalomedullary nailing. The effectiveness of dynamic hip screw fixation for pertrochanteric fractures without using traction table is not well investigated. This study aimed to find out the mean tip apex distance in patients undergoing dynamic hip screw fixation for pertrochanteric fractures without using traction table.

**Methods:**

A descriptive cross-sectional study was conducted among patients undergoing dynamic hip screw fixation for pertrochanteric fractures without using traction table between 1 September 2021 and 30 June 2022 after getting approval from institutional review committee (Reference number: IRC-2021-08-23-02). All patients undergoing dynamic hip screw fixation for pertrochanteric fractures without using traction table were included in the study. Patients with pre-existing ipsilateral or contralateral hip deformity, contra-lateral hip prosthesis, bilateral hip fractures, and history of prior ipsilateral hip surgeries were excluded. Convenience sampling was done. Point estimate and 95% confidence interval were calculated.

**Results:**

Among 45 patients, the mean tip apex distance was 20.45±6.13 mm (18.66-22.24 mm, 95% Confidence Interval). Among 45 patients, 24 (53.33%) were males and 21 (46.66%) were females. The average age of the participants was 67.75±21.33 years.

**Conclusions:**

The mean tip apex distance in patients undergoing dynamic hip Screw fixation for pertrochanteric fractures without using traction table was similar to that reported in other international studies.

## INTRODUCTION

Dynamic Hip Screw (DHS) fixation has shown to be equally effective compared to cephalomedullary nailing and has some advantages, such as low intra-operative blood loss and treatment costs.^1-3^ Hence, it has been a preferred method for treating pertrochanteric fractures in low-income countries.

Traction table has widely been used to achieve and maintain reduction during pertrochanteric fracture fixation.^4^ However, using traction table has several disadvantages, such as lengthy preparation and anesthesia time and incidence of several complications.^4^ In addition, traction table may not be available in many centers in low-income countries due to its high cost.^5^ Pertrochanteric fracture fixation without using traction table has also been practiced, especially in patients undergoing cephalomedullary nailing.^6^ Its effectiveness in patients undergoing DHS fixation has not been well investigated.

Hence, this study was conducted to find out the mean tip apex distance (TAD) in patients undergoing DHS fixation without using traction table.

## METHODS

A descriptive cross-sectional study was conducted among the patients undergoing DHS fixation for pertrochanteric fractures following the guidelines of institutional review committee (IRC) of B.&B. Hospital (Reference number: IRC-2021-08-23-02). Study duration was of 9 months (from 1 September 2021 to 30 June 2022). Written informed consent was obtained from all the patients to be included in the study. All patients undergoing DHS fixation for pertrochanteric fractures without using traction table were included. Patients with pre-existing ipsilateral or contralateral hip deformity, contra-lateral hip prosthesis, bilateral hip fractures, and history of prior ipsilateral hip surgeries were excluded.

The sample size was calculated using the following formula:


$$\begin{array}{ll} \text{n} & ={\text{Z}}^{2}\times \frac{\sigma^{2}}{{\text{e}}^{\text{2}}}\\ & =1.96^{2}\times \frac{6.1^{2}}{2^{2}}\\ & =36 \end{array}$$

Where,

n = minimum required sample size
Z = 1.96 at 95% Confidence Interval (CI)
σ = standard deviation value, 6.1^7^
e= margin of error, 2%

The calculated sample size is 36. We have included 45 patients in our study.

All patients underwent operation within 72 hours of admission under spinal or epidural anesthesia. Preoperative antibiotic prophylaxis of cefazoline 1 gm or cefuroxime 1.5 gm was given 30 minutes prior to incision. Patients were placed supine on standard radiolucent table with a bolster/sandbag under the buttock of affected side and were draped in such a way that the whole affected limb was free for the application of manual traction.

DHS was performed following AO technical manual using standard lateral incision with vastus lateralis muscle splitting technique.^8^ Standard stainless steel or titanium DHS locking plate (3-5 hole) and 7.8 mm lag screw were used depending upon the bone quality and patient's preference. An additional 6.5 mm partial or fully threaded cannulated cancellous screw was used as de-rotation screw depending upon fracture stability. Surgeries were performed by wide range of board-certified orthopedic surgeons with more than five years of clinical experience.

Closed reduction was performed by an assistant with longitudinal traction and internal rotation. In cases where closed reduction was difficult, an open reduction was done, either by directly visualizing the fracture site or by indirect manipulation using bone lever or hook. Multiple (2 or 3) 2.0 mm pins were inserted to stabilize the reduction in such a way that the pins incorporate major fracture fragments and do not obstruct the guide-wire placement. The reduction was checked under c-arm in both anteroposterior (AP) and frog-leg lateral views.^9^

Primary outcome measure was accuracy of lag screw placement as determined by post-operative TAD. Secondary outcome measures were quality of reduction (QOR) and complication rate.

TAD was measured in mm using a formula provided by Geller et al.^10^ a modification of Baumgaertner's^11^ original formula, using post-operative AP and lateral radiographs without adjusting the magnification. Patients were then grouped into two categories: TAD ≤25 mm and TAD >25 mm.


$$\text{TAD}=\left({\text{X}}_{\text{AP}}\times \frac{{\text{D}}_{\text{T}}}{{\text{D}}_{\text{AP}}}\right)+\left({\text{X}}_{\text{LAT}}\times \frac{{\text{D}}_{\text{T}}}{{\text{D}}_{\text{LAT}}}\right)$$

(X_AP_ = distance from the tip of the lag screw to the center of the femoral head in AP view; X_LAT_ = distance from the tip of the lag screw to the center of the femoral head in lateral view; D_T_ = true inner diameter of lag screw (7.8 mm); D_AP_ = inner diameter of lag screw in AP view; D_LAT_ = inner diameter of lag screw in lateral view)

QOR was evaluated using post-operative AP and lateral radiographs and classified into three groups based on Baumgaertner's criteria as: Good [if normal or slightly valgus neck-shaft alignment on the AP radiograph, <20° of angulation on the lateral and displacement of <4 mm on either view (compared to contralateral hip)], Acceptable [if the reduction met the requirement in terms of either alignment or displacement], and Poor [if reduction met neither criteria].^11^

Complication rate was defined as incidence of any technique-related complications, such as breakage of pins/guidewires and revision surgery. Revision surgery was defined as the requirement of any surgical procedure to modify the initial fixation construct following the evaluation of post-operative radiograph.

All measurements were done by principal investigator and were verified by two senior orthopedic surgeons with more than 10 years of clinical experience. Following data were extracted: age, sex, mechanism of injury, fracture type (AO classification), side of injury, operating time (duration from start of the incision to closure), TAD, post-operative QOR, and complications. Continuous data were reported as mean±standard deviation and categorical data were reported as number (percentage). Point estimate and 95% CI were calculated.

## RESULTS

The mean TAD in patients undergoing DHS fixation for pertrochanteric fracture without using traction table was 20.45±6.13 mm (18.66-22.24 mm, 95% CI). Among 45 patients, 24 (53.33%) were males and 21 (46.66%) were females. The average age of the participants was 67.75±21.33 years. The commonest mechanism of injury was trivial fall, 33 (73.33%). The commonest fracture type was AO 31A2, 30 (66.66%). Closed reduction was successful in 32 (71.11%) cases. The mean duration of operation was 76.97±21.41 minutes.

De-rotation screw was used in 12 (26.66%) cases. Among 45 patient, TAD ≤ 25 mm was observed in 35 (77.77%) cases. The QOR was good in 41 (91.11%) cases, acceptable in 4 (8.88%) and poor in 0 (0%) cases. There were no technique-related complications (Table 1).

**Table 1:** Outcomes of the study.

Characteristics	n (%)
Sex distribution	
Male	24 (53.33)
Female	21 (46.66)
Mechanism of injury	
Trivial fall	33 (73.33)
Road traffic accidents	8 (17.77)
Fall from height	3 (6.66)
Others	1 (2.22)
Fracture type	
^*^AO 31A1	12 (26.66)
^*^AO 31A2	30 (66.66)
^*^AO 31A3	3 (6.66)
Side of injury	
Right side	33 (73.33)
Left side	12 (26.66)
Duration of operation, in minutes	
Mean±SD^†^	76.97±21.41
Range	35 to 120
Method of fracture reduction	
Closed	32 (71.11)
Open	13 (28.88)
^**^TAD, in mm	
Mean±SD	20.45±6.13
Range	13.3 to 34.5
^**^TAD	
≤25 mm	35 (77.77)
>25 mm	10 (22.22)
^#^QOR	
Good	41 (91.11)
Acceptable	4 (8.88)
Poor	-

* AO Arbeitsgemeinschaft für Osteosynthesefragen

† SD standard deviation

** TAD tip apex distance

# QOR quality of reduction.

## DISCUSSION

In this study, the mean TAD in patients undergoing DHS fixation without using traction table was 20.45±6.13 mm (range, 13.3 to 34.5 mm). The findings were similar to that reported in previous studies evaluating DHS fixation without using traction table.^7,9^ A study conducted in India including 328 patients with pertrochanteric fractures who were treated with DHS fixation without using traction table observed mean TAD of 19.7±6.1 mm.^7^ Similarly, another study conducted in Malaysia observed mean TAD of 23.1 mm in 16 patients who underwent DHS fixation without using traction table.^9^ In addition, same study conducted in Malaysia observed mean TAD of 20.7 mm in patients who underwent DHS fixation with using traction table.^9^ TAD is considered to be a major predictor for screw cutout and fixation failure in patients undergoing DHS fixation and TAD ≤25 mm is considered to be the required TAD to avoid such complication.^11^ In this study, out of 45 patients, 35 (77.77%) had TAD ≤25 mm. These findings suggests that DHS fixation for pertrochanteric fracture can effectively be performed without using traction table.

In this study, good quality of reduction was observed in 41 (91.11%) cases, acceptable reduction was observed in 4 (8.88%) cases. The outcomes were better than that reported in previous study conducted in India.^7^ They observed good reduction in 212 (64.63%) cases, acceptable reduction in 106 (32.31%), and poor reduction in 10 (3.04%) cases.^7^ However, the authors acknowledged that the large number of cases with acceptable reduction was observed initially when they were not so familiar with DHS fixation without using traction table.^7^ This suggests that with better understanding of the technique good reduction can effectively achieved in patients undergoing DHS fixation without using traction table.

In this study, the mean age of the participants was 67.75±21.33 years (range, 23 to 97 years). The finding was similar to that reported in previous studies.^7,9^ The study conducted in India included participants with mean age of 68.2±11.9 years and the study conducted in Malaysia included participants with mean age of 73 years (range, 22 to 92 years).^7,9^ This suggests that pertrochanteric fractures are common in the elderly. Among 45 patients, 24 (53.33%) were males and 21 (41.66%) were females. It is known from the literature that pertrochanteric fractures are common among females, with ratio of 2:1.^2^ The reason behind male predominance in this study could be due to smaller sample size and increased level of outdoor activity among males in our country. Trivial fall from standing height was the most common mechanism of injury, in 33 (73.33%) cases. This finding was well supported by the literature that pertrochanteric fractures often results from trivial trauma in the elderly.^2,9^

In the study, DHS fixation was performed in all types of pertrochanteric fractures, and majority of them were AO31 A1 and A2 types. Although cephalomedullary nails have gained popularity for the treatment of pertrochanteric fractures, some previous studies have found no significant benefit of cephalomedullary nailing when compared to DHS in treating patients with AO31 A1 and A2 pertrochanteric fractures.^1,3^ As treatment cost is an important issue in low-income countries, DHS fixation remains a choice of fixation for AO31 A1 and A2 type fractures. However, it is now established in the literature that cephalomedullary nails are biomechanically superior to DHS fixation in treating patients with unstable pertrochanteric fracturs, i.e. AO31 A3 types.^12^ We also agree to that fact that cephalomedullary nailing would have been a better choice of fixation in those patients. However, 3 (6.66%) cases underwent DHS fixation for AO31 A3 type fracture because of cost issues.

The mean duration of operation was 76.97±21.41 minutes (range 35 to 120 minutes), which was higher than that reported in previous studies conducted in India (37±22 minutes) and Malaysia (54.6 minutes).^7,11^ The longer duration of operation could be due to the practice of obtaining X-rays before wound closure in our hospital, which often takes 10-15 minutes. However, the duration of operation was similar to what reported in the study conducted in Iran, which reported mean duration of 78.7±14.8 minutes.^13^ In addition, the study conducted in Malaysia observed the mean duration of operation of 57 minutes with using traction table.

DHS fixation without using traction table is not devoid of challenges. Achieving adequate reduction is a major concern.^7,9,13^ However, in majority of the cases 32 (71.11%), closed reduction was successful. Obtaining lateral c-arm images is another difficulty.^7,9,13^ However, in this study, frog-leg lateral images were obtained as described in previous study conducted in Malaysia.^9^ Similarly, bending of provisional reduction pins or loss of reduction while obtaining frog-leg lateral images are other concerns.^7^ However, previous studies have suggested that inserting 2-3 reduction pins and careful manipulation while obtaining frog-leg lateral views could prevent such complications.^9,13^ In this study, no such complications were observed. This suggests that DHS fixation can effectively be performed without using traction table.

This study has some limitations. The study outcomes are only applicable to those undergoing DHS fixation for pertrochanteric fractures without using traction table. The effectiveness of this technique over DHS fixation using the traction table is not evaluated. Hence, further research is warranted. Similarly, patients were not followed-up to evaluate fracture healing and screw cut-out rate, because of inherent difficulties. This suggests that long-term efficacy of this technique is not clear. However, previous studies have shown that all fractures united within mean duration of 1214 weeks and no screw cut-out was observed within mean follow-up of 31±23 months.^9,13^ Furthermore, all the measurements were done by principle investigator manually, suggesting the risk of measurement bias. However, the measurements were verified by two senior consultants to minimize the bias.

## CONCLUSIONS

The mean TAD in patients undergoing DHS fixation for pertrochanteric fractures without using traction table was similar to that reported in other international studies. The QOR achieved was better compared to that reported in other international studies. However, the mean duration of operation was longer than that reported in other international studies.
